# A Rare Case of Chest Wall Abscess by Nocardia in a Patient With Sarcoidosis

**DOI:** 10.7759/cureus.26769

**Published:** 2022-07-12

**Authors:** Mahreen Sana, Faheem Mahmood Butt, Muhammad Imran ul Hasan, Adnan Amir

**Affiliations:** 1 Pulmonology, Shaukat Khanum Memorial Cancer Hospital and Research Centre, Lahore, PAK

**Keywords:** co-trimoxazole, immunosuppressants, sarcoidosis, nocardia, chest wall abscess

## Abstract

Nocardia is a rare gram-positive pathogen reported to cause infections in immunocompromised individuals. It usually involves the lungs but may also lead to abscess formation; cases of disseminated nocardiosis have also been reported. We are presenting a case of an Asian male who had sarcoidosis with pulmonary and skin involvement. The patient was on long-term immunosuppressive therapy with corticosteroids with good control of the disease. He developed a fever, weight loss, and right-sided chest pain. CT of the chest showed new nodular infiltrates. Worsening of sarcoidosis was suspected; the corticosteroid dose was increased and methotrexate was started. There was no favorable response to the increase in immunosuppressive therapy. Weight loss was followed by worsening shortness of breath and fluctuant swelling in the right lateral half of the chest. Bronchoalveolar lavage was done to rule out tuberculosis but it did not show any organism’s growth. Ultrasound-guided needle aspiration from the abscess was done that showed growth of Nocardia species. Therapeutic dose co-trimoxazole (trimethoprim-sulphamethoxazole) was started as first-line therapy after confirming the organism’s drug susceptibility pattern along with needle aspiration of the collection on the chest wall. Immunosuppressive agents were stopped. There was a good response to treatment with resolution of symptoms within two months. However, complete radiological recovery took 10 months. Co-trimoxazole (trimethoprim-sulphamethoxazole) therapy continued for two months after radiological recovery. Physicians, therefore, should keep Nocardia as an important differential diagnosis while treating the immunosuppressed population.

## Introduction

Nocardiosis is an uncommon disease caused by Nocardia species, which are gram-positive, aerobic, branching, weakly acid-fast, actinomycetes found in soil, and decaying matter [1]. The disease is seen in immunosuppressed patients like those with human immune deficiency virus (HIV), on corticosteroids, other immunosuppressive agents, and solid organ transplant recipients [2]. It has an incidence rate of 500-1000 cases per year in the United States, with males affected more than females [3]. Lung involvement is seen in 70-75% of patients with nocardiosis [4]. However, the disease may become disseminated and may involve other organs [5]. Secondary hematogenous involvement of the chest wall is a rare occurrence, and only a few cases have been reported worldwide [6]. 

## Case presentation

This is a case report of a 45-year-old Asian male patient with a diagnosed case of sarcoidosis with pulmonary and skin involvement. He had been on steroids for the past four years with dose adjustments according to symptoms and had steroid-induced diabetes and hypertension. He presented with right-sided chest pain, weight loss, anorexia, and fever for one month. He had good control of symptoms on 10 mg/day of prednisolone. On initial workup, CT of the chest showed worsening right-sided nodular opacities that were thought to be related to worsening sarcoidosis. The infectious profile, including cultures and mycobacterial GeneXpert of the sputum, turned out to be negative. He was started on methotrexate 10 mg/week and the dose of corticosteroids was increased to 40 mg/day.

Despite treatment, he started experiencing worsening cough, further weight loss, shortness of breath on exertion, and swelling on the right side of his chest. On examination, he had a temperature of 37.8°C, a pulse rate of 96/minute, blood pressure of 150/90 mm Hg, and a respiratory rate of 22/minute. There was a right upper back swelling that was soft in consistency and fluctuant, with normal overlying skin. It was non-tender; according to him, he fell a few weeks ago and injured his back. On auscultation of the chest, there were decreased breath sounds on the right side.

**Figure 1:**
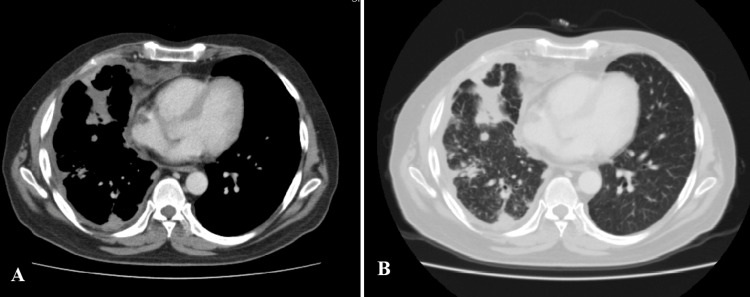
(A) CT chest with contrast showing the collection in and beyond the thoracic cage. (B) CT chest with contrast (lung parenchymal window) showing consolidation in the right upper lobe and nodular infiltrates.

His total leukocyte count was elevated to 13,000/mm^3^ with 87.9% neutrophils. A chest x-ray showed worsening right upper lobe consolidation. CT thorax (Figure 1A, 1B) showed further worsening of the disease in the right lung with innumerable peripherally enhancing fluid collections in the right pleural space with extension beyond the chest wall into the right thoracic muscles; multiple nodules in the right lung and fibrosis in the right upper lobe. Differential diagnoses included tuberculosis and nocardiosis. 

**Figure 2:**
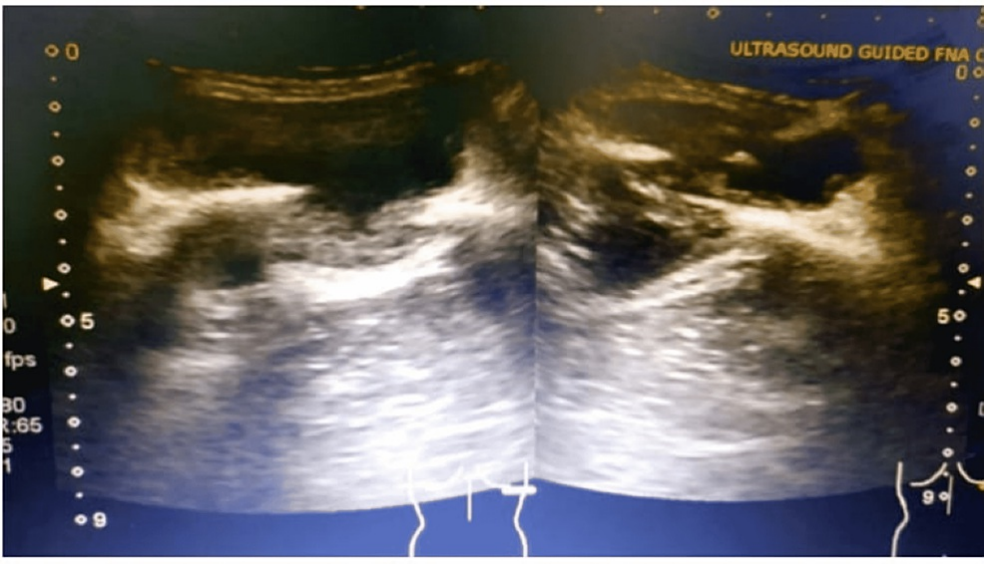
Ultrasound-guided aspiration of the right rib cage collection.

His bronchoscopy and bronchoalveolar lavage were done. All pan cultures, including fungal, bacterial, mycobacterial, and mycobacterial GeneXpert, were negative. Ultrasound-guided aspiration of chest wall swelling was done. Aspiration of swelling was done under ultrasound guidance (Figure 2). A 25 ml thick purulent material was aspirated and sent for cultures. The culture of the aspirate showed growth of Nocardia species after two weeks. The patient was started on co-trimoxazole (trimethoprim-sulphamethoxazole) (TMP-SMX) after confirming the drug susceptibility of the organism. However, we were not able to identify species due to a lack of resources. Steroids and methotrexate were stopped as sarcoidosis was in remission. 

**Figure 3:**
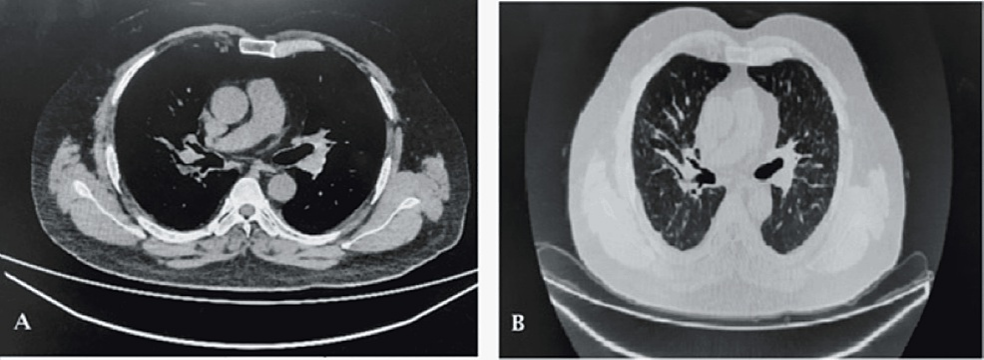
(A) CT thorax showing resolution of the right rib cage collection. (B) CT thorax (lung parenchymal window) showing resolution of consolidation and nodularity.

There was a good response to antibiotic therapy. His fever resolved, the swelling subsided, and he started gaining weight over two months. However, complete radiological improvement took 10 months. CT thorax repeated after 10 months of treatment showed resolution of right perihilar consolidation and bilateral diffuse nodularity (Figure 3). Treatment continued for two months after the resolution of radiological findings. Sarcoidosis remained in remission. 

## Discussion

Nocardia is a rare and increasingly important opportunistic pathogen in immunosuppressed individuals. Many species of Nocardia have been reported, however around nine species are known to cause disease in humans, including *N. asteroides*, *N. brasiliensis*, *N. otitidiscaviarum*, *N. farcinica*, *N. abscessus*, *N. nova*, *N. transvalensis*, *N. pseudobrasiliensis*, and *N. africana*. It can be introduced by trauma to the skin or by inhalation. A single organ can be involved or the disease may become disseminated, with the invasion of the nearby blood vessels. Lungs are the most common site of infection. Pulmonary infection can travel through the chest wall and result in chest wall abscesses. It can involve any organ and system, including the brain, kidneys, skin, joints, and subcutaneous tissue. Extrapulmonary infections without lung involvement can also occur. Pyogenic abscesses are also reported by Nocardia. Prognosis is poor when there is central nervous system involvement [7].

Prompt diagnosis and treatment are crucial for patients with nocardiosis. Diagnosis of Nocardia is often delayed due to the rarity of the disease and co-infections. Diagnosis of Nocardia is also challenging due to non-specific symptoms, clinical findings, and its similarity with other respiratory pathogens. Mycobacterium tuberculosis also presents features like pulmonary nocardiosis, such as fever, weight loss, cough, and pneumonia. Whenever Nocardia is suspected, the laboratory should be informed to use special staining techniques and culture media to isolate Nocardia. Sputum smears are often negative, and it takes two to four weeks to grow on special agar culture media. A modified acid-fast stain with 1% sulfuric acid is used to identify Nocardia species where the bacilli are seen as pink-colored filaments [8,9].

Human nocardiosis is underestimated due to the limited data available, mostly as case reports. Early identification, a high index of suspicion, and appropriate antibiotics are needed to improve outcomes in patients with Nocardiosis. Mortality increases with advanced immunocompromised state, CNS involvement, solid organ transplant recipients, delay in starting treatment, dissemination of disease with multi-organ involvement, and early discontinuation of antibiotics. Recurrence is also seen in patients with advanced immunocompromised states, where antibiotics are stopped early [10,11]. Overall mortality from Nocardia is reported to be up to 19-22% [12]. 

Treatment of Nocardia should be based on susceptibility patterns. Sulfonamides, including sulfadiazine and sulfisoxazole, have been used to treat Nocardia for the past 50 years. Co-trimoxazole (trimethoprim-sulphamethoxazole) is the most used preparation. It is used in divided doses of 5 to 10 mg/kg per day of the trimethoprim component (or 25 to 50 mg/kg per day of sulphamethoxazole). The drug is active against most species of Nocardia. However, *N. otitidiscaviarum* is found to be resistant [13]. Alternative agents include amikacin, imipenem, meropenem, ceftriaxone, cefotaxime, minocycline, moxifloxacin, levofloxacin, linezolid, tigecycline, and amoxicillin-clavulanic acid. These agents can be used as the first line for those who have sulfonamide allergy or resistance, or as an adjunct to TMP-SMX in those with CNS involvement or disseminated infection. Imipenem and minocycline have good activity against Nocardia. Linezolid has good coverage against all Nocardia species. It is particularly useful when the disease is disseminated or when there is CNS involvement. Amoxicillin-clavulanic acid has moderate activity against Nocardia. A few species of Nocardia are reported to have a higher degree of resistance, which include *N. farcinica*, *N. brasiliensis*, and *N. otitidiscaviarum*. The occurrence of any side effects of the used drugs should be monitored at regular intervals [13,14]. Treatment should be for a longer duration to prevent the recurrence of the disease. It is advised to continue treatment for a total of 6-12 months or two to three months after the resolution of radiological findings [15]. Our patient had chest wall involvement along with lung involvement and responded well to one-year treatment of co-trimoxazole based on the organism’s drug susceptibility pattern. 

## Conclusions

Nocardia is a rare but important opportunistic pathogen for immunocompromised individuals. It is important for clinicians, treating such patients, to keep a high index of suspicion for Nocardia. The infection most commonly involves the lungs and, if delayed, can become disseminated, which can result in a poor prognosis. Early diagnosis and prompt treatment improve survival and give favorable outcomes in patients with nocardiosis.
